# Posterior reversible encephalopathy syndrome (PRES) and CT perfusion changes

**DOI:** 10.1186/1865-1380-5-12

**Published:** 2012-02-29

**Authors:** Vishnumurthy Shushrutha Hedna, Latha Ganti Stead, Sharathchandra Bidari, Akhil Patel, Amareshwari Gottipati, Christopher G Favilla, Arash Salardini, Aunali Khaku, Diana Mora, Ajay Pandey, Het Patel, Michael F Waters

**Affiliations:** 1 Department of Vascular Neurology, University of Florida, Archer Road, Gainesville, Florida, 32611, USA; 2 Department of Emergency Medicine, University of Florida, Archer Road, Gainesville, Florida, 32611, USA; 3 Department of Neuro-Radiology, University of Florida, Archer Road, Gainesville, Florida, 32611, USA

## Abstract

Posterior reversible encephalopathy syndrome **(**PRES) can present with focal neurologic deficits, mimicking a stroke and can often represent a diagnostic challenge when presenting atypically. A high degree of suspicion is required in the clinical setting in order to yield the diagnosis. Cerebral CT perfusion (CTP) is utilized in many institutions as the first line in acute stroke imaging. CTP has proved to be a very sensitive measure of cerebral blood flow dynamics, most commonly employed to delineate the infarcted tissue from penumbra (at-risk tissue) in ischemic strokes. But abnormal CTP is also seen in stroke mimics such as seizures, hypoglycemia, tumors, migraines and PRES. In this article we describe a case of PRES in an elderly bone marrow transplant recipient who presented with focal neurological deficits concerning for a cerebrovascular accident. CTP played a pivotal role in the diagnosis and initiation of appropriate management. We also briefly discuss the pathophysiology of PRES.

## Background

Posterior reversible encephalopathy syndrome (PRES), as the name suggests, is a constellation of symptoms caused by reversible ischemia most commonly of the posterior cerebral vasculature, thus affecting the parietal-occipital region. Still other vascular territories can be affected in PRES (see Table 1).Various terminologies have been used to describe this condition, including "reversible posterior leukoencephalopathy syndrome" and "reversible posterior cerebral edema syndrome" among others [1]. Hypertension (HTN) is the most commonly identified cause of PRES, followed by medications, eclampsia and systemic factors. The pathophysiology of HTN related PRES is due to a failure of cerebrovascular autoregulation, which in turn results in vasogenic edema. Non-hypertensive PRES may be due to an autoimmune or immune response to various stimuli [2]. The pathology usually affects the posterior brain hemisphere (parietal-occipital region), which may be a consequence of reduced sympathetic innervation in this area. Usually it is a reversible phenomenon, as indicated by the name, but if not recognized early and treated appropriately, permanent brain injury may ensue.

**Table 1:** Common location of PRES

Common location of PRES
Parietal-occipital - most common.
Posterior frontal
Temporal
Thalamus
Cerebellum
Brainstem
Basal ganglia

## Case presentation

**Figure 1:**
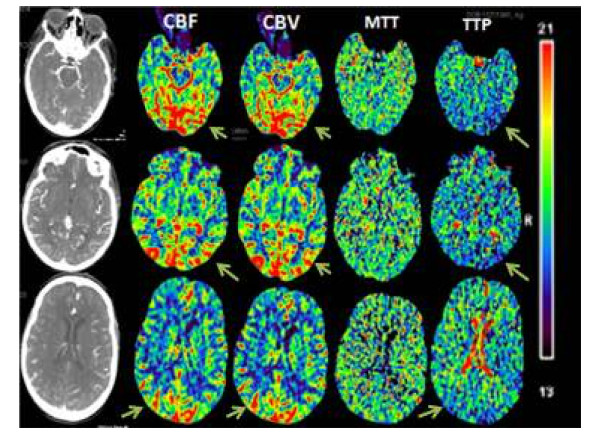
**CTP images in our case**.

**Figure 2:**
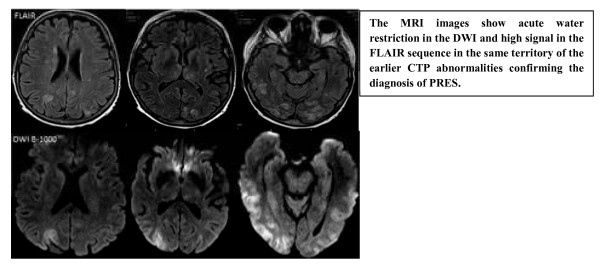
**MRI images in PRES**.

**Figure 3:**
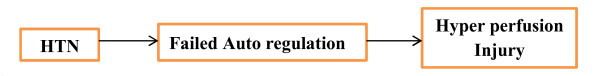
**Most commonly attributed theory of PRES**.

A 70-year-old white female presented to the emergency room with symptoms of a cerebrovascular accident. She had a history of multiple myeloma status post-autologous bone marrow transplant (BMT) with a conditioning regimen of high-dose melphalan 2 weeks prior to presentation. She woke up the morning of presentation and was found to be confused for a few minutes, followed by a gradual improvement in mental status. About an hour later, she started to experience a severe headache associated with blurry vision, and shortly thereafter she became disoriented again. Paramedics identified agitation, right-side neglect, left gaze deviation and right side weakness. On arrival in the emergency department, the patient's headache had resolved, but the patient was still agitated and disoriented. The patient's altered mental status (AMS) required that the history be obtained from the patient's husband. There was no history of recent infection, fever, weight loss or trauma. The review of systems was negative for photophobia, seizures or any other neurological issues. Pertinent past medical history was that of recent BMT with melphalan and poorly controlled hypertension. She had had thrombocytopenia since the time of BMT and chemotherapy. Her admission blood pressure was 221/114 with a mean arterial pressure (MAP) of 145 mmHg. Her admission NIH stroke scale score was 7, with problems in orientation, not following commands, not answering questions appropriately, left gaze preference, reduced blink on stimulus from the right and possible right-sided neglect. Her visual acuity was reduced to finger movements and light perception in both eyes. She was moving her extremities equally, bilaterally. Reflexes were brisk throughout with equivocal plantar response. The rest of the neurological exam was limited, as the patient was not following commands consistently. Our differential diagnosis at that time included cerebrovascular accident (CVA), PRES (due to elevated BP, recent chemotherapy and bone marrow transplant), seizures and complicated migraine. Since there was no motor deficit associated with the neglect and eye deviation, we were obligated to consider a broad differential diagnosis, including PRES. After the initial laboratory workup, we obtained a CT head and a CT angiogram of the head and neck with perfusion studies. The CTA of the head and neck failed to identify any major vessel cutoff or any acute hypo/hyper density, but the CTP demonstrated increased cerebral blood volume (CBV), cerebral blood flow (CBF) and reduced time to peak (TTP) in the posterior cerebral vascular distribution (see Figures 1 and 2). These imaging features were consistent with PRES, and we initiated intravenous anti-hypertensive medications. An MRI brain was obtained, which showed abnormal restriction in the parietal and occipital areas, confirming the diagnosis of PRES (see Figure 3). Reduction of the patient's systolic BP from 220 to 180 was associated with slight improvement in her visual acuity and orientation within a couple of hours. Notable laboratory data revealed a platelet count of 11,000/μl and hemoglobin of 11 g/dl. All other laboratory tests were within the normal limits. There was no evidence of thrombotic thrombocytopenic purpura (one of the causes of PRES), and she was admitted with the diagnosis of PRES. At the time of admission, PRES was considered to be secondary to malignant hypertension complicated by recent chemotherapy and BMT. Over the subsequent 48 h, she returned to baseline with improvement of her blood pressure to normal range.

## Discussion

PRES commonly presents with seizures (74%), altered sensorium or encephalopathy, headache and visual changes [3]. Other neurological features such as aphasia and sensory changes are also seen. PRES can sometimes present similarly to CVA, such as in our case. In such cases, the patient may inadvertently and inappropriately receive thrombolytic therapy. We again stress the point that when a patient presents with stroke-like symptoms, but with an inconsistent neurological exam, then the stroke mimics such as PRES, seizures, migraine and tumor should be included in the differential diagnosis. The etiology of PRES can be broadly divided into five main etiological groups. In order of clinical frequency, PRES etiologies include HTN (61%), cytotoxic medications (19%), preeclampsia or eclampsia (6%) [3], autoimmune and systemic conditions, including sepsis (see Table 2). Currently, the pathophysiology of PRES is controversial. The most accepted theory of HTN-related PRES is that of a hyperperfusion injury model (see Figure 3). There will be a failure of cerebral autoregulation in relation to the sudden elevation of blood pressure. This sudden increase in MAP can lead to arteriolar dilation, hyperperfusion, endothelial vascular damage and disruption of the blood brain barrier. This leads to vasogenic edema and potentially reversible ischemia affecting both the grey and white matter [4]. Another less accepted theory argues that HTN-related dysautoregulation results in vasoconstriction and hypoperfusion injury. However, the above hypothesis fails to explain non-hypertensive PRES, and so some postulate an autoimmune or immune response theory to various stimuli [2].

**Table 2:** Etiology of PRES

Common causes of PRES
**H**ypertension
**E**clampsia and preeclampsia
**D**rugs:
- Recreational: Cocaine, Amphetamines, PCP, LSD
- Others: Anti-depressants (Tricyclics, MAO Inhibitors), Bronchodilators, Erythropoietin, Midodrine, Fludrocortisone, Triple H therapy, Intravenous immunoglobulins (IVIG).
**N**eoplastic drugs: Cyclosporine-A, Tacrolimus, Interferons, Indinavir, Cisplatin, Cytarabine, Gemcitabine.
**A**utoimmune and **S**ystemic: Systemic lupus erythematosus (SLE), Scleroderma, Vasculitis like PAN, Wegener's, Thrombotic thrombocytopenic purpura (TTP), Henoch-Schönlein purpura, Hemolytic uremic syndrome (HUS), Amyloid angiopathy, Tumor lysis syndrome, Systemic inflammatory response syndrome (SIRS), Sepsis, Multiple organ Dysfunction, Electrolyte imbalance (Hypomagnesemia, hypercalcemia), Hypocholesterolemia,, GBS, Head injury, Renal failure due to any etiology.

We suggest that PRES is the result of various etiological factors that lead to blood brain barrier injury either by hyper- or hypoperfusion, endothelial dysfunction, changes in blood vessel morphology, hypocapnia or immune system activation [2,4,5]. It usually affects parietal and occipital area, s but other regions can be involved as well [6].

In our patient we feel that elevated MAP led to regional dysautoregulation, consequently causing hyperperfusion, explaining the findings of increased CBF, CBV and reduced TTP. It is important to recognize that this patient had a recent history of bone marrow transplant with exposure to chemotherapy, which may also be a causative factor in the development of PRES, or may have independently contributed to the tissue injury.

We conclude that the mechanism of PRES is individualized in each patient and depends mainly on the causative factor identified in each case. In the setting of HTN, PRES is most likely due to the mechanism described in the previously discussed theories. In the setting of normotensive PRES, the mechanism may be based on endothelial dysfunction, immune system activation and other systemic features. Although initially edema is vasogenic in nature, a failure to reverse the disease etiology will subsequently cause cytotoxic edema and eventually brain infarction, further emphasizing the importance of early disease recognition.

A high degree of suspicion is required to make this diagnosis as the patient may present atypically. Since many patients with PRES present with inadequate history and AMS, brain imaging plays an important role in the diagnosis of PRES. Even though MRI (particularly T2-FLAIR) is the imaging of choice, CTP can play a significant role that can reveal the cerebral hemodynamics related with this condition. Since there is hyperperfusion the CBF and CBV will be elevated, and TTP will be reduced. The CTP can also have diametrically opposite findings compared to our case [7] (see Table 3). The brain MRI changes on FLAIR with no changes on diffusion weighted images (DWI) suggest vasogenic edema. Changes in both sequences (FLAIR and DWI) indicate that the cytotoxic edema has set in, which may have an unfavorable outcome. The authors imply that CTP is useful but not superior to MRI in the diagnosis of PRES.

**Table 3:** CT perfusion changes in various brain insults

Etiology	CBF	CBV	MTT	TTP
Infarct core	↓	↓	↑	↑

Ischemic penumbra	Varies; usually↓	↑	Varies; usually ↑	↑

seizure	↑	↑	↓	↓

PRES*	Usually ↑ but can be ↓	↑ or ↓	Equivocal	↓

CBF = cerebral blood flow; CBV = cerebral blood volume; MTT = mean transit time; TTP = time to peak.

*= CTP changes in PRES depend on whether it is a hyperperfusion or hypoperfusion mechanism of PRES.

The management of this condition depends on the etiology and should be initiated in a timely manner. The treatment of the underlying cause is typically sufficient to reverse this condition. However, a word of caution: this condition can lead to irreversible brain insult if the treatment is delayed or if there is prolonged brain insult, in which case a brain insult then becomes irreversible brain infarctions. Additionally, there may be hemorrhagic complications and raised intracranial pressure contributing further to the cerebral damage [8]. The MAP should be reduced quickly but with caution in the cases of hypertensive PRES. In cases of non-hypertensive PRES, especially in case of neoplastic drugs, the offending agent should be withdrawn quickly to avoid further damage to the blood brain barrier. In the setting of transplant, alternative medications can be substituted to avoid organ rejections [9]. If an autoimmune etiology is suspected, then immunosuppression has a role in the management [10]. Seizures are managed with anti-epileptic medications.

## Conclusions

Posterior reversible encephalopathy syndrome is a relatively rare syndrome that sometimes presents as a stroke mimic. As such, it is important for the emergency physician to recognize. Urgent recognition and early initiation of management of this condition are imperative as it directly impacts the neurological outcome. Brain CT perfusion can play an important role in the diagnosis.

## Consent

Written informed consent was obtained from the patient for publication of this case report and any accompanying images. A copy of the written consent is available for review by the Editor-in-Chief of this journal.

## Competing interests

The authors declare that they have no competing interests.

## Authors' contributions

All the authors were involved in the draft, graphics, grammar correction, creation of tables and figures. All authors read and approved the final manuscript.
